# The two-component regulator CvsR has a small core regulon *in planta* and modulates *Pseudomonas syringae* global gene expression with some overlap to the pattern-triggered immunity stimulon response

**DOI:** 10.1128/spectrum.04124-25

**Published:** 2026-06-22

**Authors:** Hsiao-Chun Chen, Carter J. Newton, Li Yang, Brian H. Kvitko

**Affiliations:** 1 Department of Plant Pathology, University of Georgia, Athens, Georgia, USA; 2 The Plant Center, University of Georgia, Athens, Georgia, USA; Pennsylvania State University, University Park, Pennsylvania, USA

**Keywords:** pattern-triggered immunity (PTI), *Pseudomonas syringae *pv. *tomato *DC3000, two-component system, *in planta *transcriptome

## Abstract

Pattern-triggered immunity (PTI) provides broad-spectrum protection in plants by activating defense responses upon perception of conserved microbial signatures such as bacterial flagellin. *In vitro* transcriptome profiling revealed that the *Pseudomonas syringae* pv. *tomato* DC3000 two-component regulator CvsR mirrors some of the broader regulatory patterns observed under the exposure to PTI *in planta*. Our analyses indicated that during infection *in planta*, CvsR primarily governs a small core regulon centered on carbonic anhydrase and its associated transporter. Comparative RNA-seq analyses between the Δ*cvsR* and wild-type strain further confirm this narrow regulatory scope. Moreover, the majority of bacterial transcriptional shifts appear to reflect indirect consequences of response to the host immune environment rather than direct CvsR-dependent regulation, including responses associated with sulfate starvation. Together, these findings suggest that PTI-driven bacterial transcriptional reprogramming is shaped predominantly by host immune status, with CvsR exerting modest, targeted control restricted to a limited set of genes.

**IMPORTANCE:**

Pattern-triggered immunity (PTI) provides broad-spectrum disease resistance in plants by recognizing conserved microbial patterns such as bacterial flagellin. Activation of PTI alters the environment that pathogens encounter during infection, yet how bacteria respond to these immune-imposed conditions at the molecular level remains poorly understood. In this study, we profile the bacterial transcriptome directly in planta during infection with RNA-seq, providing a detailed view of pathogen responses under immune pressure. We focus on the previously identified two-component regulator CvsR and show that, despite widespread transcriptional changes induced by PTI, CvsR directly controls only a small core set of genes in planta. Instead, most bacterial transcriptional shifts reflect indirect responses to the immune-modified host environment. By capturing pathogen gene expression during infection, this work clarifies how plant immunity constrains bacterial physiology and provides insight that can inform sustainable strategies for crop protection.

## INTRODUCTION

Plants rely on a tiered innate immune system network to fend off pathogen attacks. Pattern-triggered immunity (PTI) and effector-triggered immunity (ETI) represent two functionally interdependent tiers of the plant immune systems (1, 2). PTI is activated when cell-surface pattern recognition receptors (PRRs) directly bind to the conserved microbial molecules, such as peptide epitopes of bacterial flagellin, elongation factor Tu, or cold shock proteins (3–6). Pathogens secrete specialized effector proteins that can suppress PTI or interfere with plant physiological responses. Resistant hosts harbor intracellular nucleotide-binding domain, leucine-rich-repeat receptors (NLRs) to recognize effectors directly or indirectly to trigger ETI. Core PTI signaling elements contribute to effective ETI, and ETI in turn enhances and sustains expression of PTI-associated genes (7). PRRs are typically ligand specific and form complexes with their co-receptors and trigger multiple signaling pathways including the mitogen-activated protein kinase (MAPK) signaling cascade, calcium-dependent protein kinases pathways, and reactive oxygen species (ROS) (2). Pre-activation of PTI by flg22 peptide 12 to 24 h prior to bacterial infiltration results in potent immune outcomes by limiting bacterial infections, effector delivery, and proliferation (8, 9). Upon activation of PTI, the apoplast undergoes rapid immune remodeling, including the accumulation of antimicrobial proteins and secondary metabolites (10–14). Additionally, PTI induces apoplast alkalization and alters ionic composition, further altering the physicochemical environment (15–17). These immune-driven changes are modeled to create a hostile microenvironment, requiring pathogens to deploy adaptive mechanisms for survival.

The bacterial pathogen *Pseudomonas syringae* pv. *tomato* DC3000 (*Pto* DC3000) is one of the most extensively studied model pathogens in plant–microbe interactions, largely due to its ability to infect the model plant *Arabidopsis thaliana* (18). Virulence determinants of *Pto* DC3000, including the type III secretion system (T3SS), its repertoire of 36 effector proteins, and the phytotoxin coronatine (19, 20), have been characterized in detail, establishing this strain as an ideal target for studying bacterial responses to PTI (21, 22). RNA-seq, in particular, has overcome prior limitations by enabling deep, unbiased detection of both abundant and rare transcripts (23–25).

While nutrient sequestration has been proposed as a component of PTI, direct evidence linking competition for these elements to bacterial growth inhibition remains limited. These studies suggest that bacterial survival during immune activation may depend on the ability to sense and respond to host-induced environmental shifts, triggering adaptive transcriptional programs. Two-component systems (TCSs) are widespread in Gram-negative bacteria, functioning as core signaling modules that coordinate responses to environmental stimuli. A canonical TCS includes a membrane-bound histidine kinase (HK) and a cytoplasmic response regulator (RR) that modulates gene expression upon phosphorylation. In *P. syringae*, the genomes encode over 60 TCS signaling modules (26). TCSs like PhoPQ, GacSA, RhpRS, CorRS, and CbrAB contribute to virulence and fitness *in planta* (27–31). The CvsRS system has been linked to transcriptional responses also seen in response to pattern-triggered immunity (PTI) (29, 32). Mutation of *cvsR* in *Pto* DC3000 shows reduced colonization and virulence in tomato and *A. thaliana*, along with downregulation of type III secretion system genes, altered motility gene expression, and increased expression of organosulfur uptake genes (29). These changes mirror transcriptional trends in PTI-exposed conditions of *Pto* DC3000 (32). To better understand the contribution of CvsR to bacterial gene regulation during host immune activation, we analyzed the CvsR regulon with and without exposure to PTI. In this study, we perform RNA-seq on wild-type *Pto* DC3000 and *ΔcvsR* under PTI-pre-activated and naïve apoplast conditions in *A. thaliana* to dissect the regulatory role of CvsR in bacterial response to PTI.

## RESULTS AND DISCUSSION

### Wild-type and *cvsR* mutant strains showed similar transcriptome profiles *in planta*

**Fig 1:**
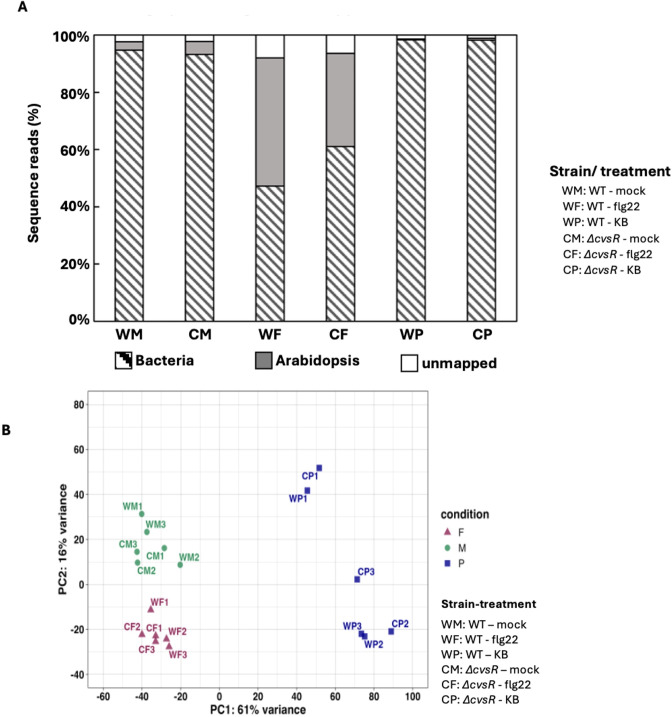
*Pseudomonas syringae* pv. *tomato* DC3000 (*Pto* DC3000) transcriptome profile in naïve and pattern-triggered immunity (PTI) condition of *Arabidopsis thaliana* leaves (**A**) The proportions of sequencing reads mapped to *Pto* DC3000 genome, the *A. thaliana* genome, and unmapped reads are shown for all samples. Total RNA was extracted from leaves pretreated to induce PTI or from untreated naïve leaves, followed by inoculation with either the wild-type (WT) or *δcvsr* mutant strain of *Pto* DC3000 at 5 h post inoculation (hpi) (*n* = 3). Additional RNA was isolated from bacterial starting inocula from King’s B medium for both WT and *δcvsr* strains (*n* = 3). (**B**) *Pto* DC3000 transcriptome profile in *A. thaliana* at 5 h post inoculation. Principal components analysis (PCA) of the gene-expression profile, measured in log-transformed bacterial gene counts (*n* = 3). Bacterial gene counts from sequenced total RNA samples inoculated pattern-triggered immunity-induced *A. thaliana* leaves (F: flg22) and inoculated naïve *A. thaliana* leaves (M:mock); *in vitro* bacterial culture from King’s B media (P).

Early-infection transcriptome profiling is difficult, as pathogen RNA represents only a minor proportion of the total RNA extracted from host tissues. However, recent advances in tissue isolation methods, library preparation strategies, and sequencing technologies have improved the ability to capture high-quality bacterial transcripts from infected plant tissues (12, 32, 33). Physical separation of bacterial cells prior to RNA extraction produced a consistently high proportion of reads mapping to the bacterial genome (32, 34). To assess how CvsR modulates *Pto* DC3000 transcription during PTI, we performed *in planta* RNA-seq on bacteria isolated from Arabidopsis leaves pretreated with flg22 or mock solution. Bacterial cells were enriched from infected tissues, and rRNA depletion of both plant and bacterial RNA substantially improved recovery of bacterial transcripts, yielding high bacterial read-mapping rates across libraries. This approach, adapted from previous protocols from Lovelace et al. (32), enabled robust detection of bacterial gene expression early during infection, including under PTI-activated conditions. Using this approach, 40%–97% of non-rRNA reads mapped to the *Pto* DC3000 genome across *in planta* libraries. On average, mock (naïve) “M” samples showed the higher mapping rate (93.5%) than flg22-pretreated PTI “F” samples (54%), while *in vitro* (pellet) “P” samples yielded 98.3% mapping (Fig. 1A; Table S1). Mapping rates to both bacterial and plant genomes were consistent across biological replicates. The reduced bacterial transcript mapping rate in PTI tissue relative to naïve tissue aligns with observations from Lovelace et al. (32). The decrease in bacterial recovery at 5 hpi in PTI conditions compared with naïve tissue likely explains this effect (26) and in our result (Fig. S1). Compared with the previous study in (32), the removal of plant rRNA was implemented and resulted in substantially higher bacterial mRNA portioned in the total detected transcripts. This modification represents the highest mapping efficiency reported to date for *in planta* bacterial transcriptomic studies (32–34) and holds potential promise for the transcriptome or genomic study of difficult patho-systems, such as *Xylella* spp., Candidatus *Liberibacter* asiaticus (CLas), or Phytoplasma (35, 36). However, physical separation of bacterial cells requires the use of structurally intact leaf tissue, as the apoplastic extraction procedure is particularly sensitive to mechanical disruption, especially during vacuum infiltration with the RNA-stabilizing buffer containing high salt concentrations. Consequently, this method may not be suitable for experimental contexts involving extensive tissue damage, such as during effector-triggered immunity (ETI) or disease progression at the late infection stage. Together, these results demonstrate that optimized host rRNA depletion and careful bacterial enrichment enable reliable transcriptomic profiling of bacterial populations within plant tissues, even under immunity-activated states.

Principal component analysis (PCA) of normalized bacterial gene counts by DESeq2 analysis revealed that *in vitro* “P” samples for both wild-type and Δ*cvsR* grown in KB medium were clearly distinct from *in planta* transcriptomes, suggesting a marked shift in bacterial gene expression between rich medium and the host environment (Fig. 1B). Within the *in planta* samples, host immune status was the primary driver of transcriptional divergence: flg22-pretreated “F” samples clustered separately from mock (naïve) “M” samples, reflecting strong transcriptional reprogramming of bacteria in response to PTI. In contrast, wild-type and *cvsR* mutant samples did not exhibit clear separation along the principal components and similar pattern was observed by sample distance clustering (Fig. 1B; Fig. S2), suggesting that host immunity exerts a stronger effect on bacterial transcriptional variation than the genetic factor of *cvsR* under these conditions. These global patterns (such as induction of motility genes) were consistent with previous *in planta* transcriptome studies from multiple laboratories, despite minor differences in duration of flg22 pre-treatment and bacterial inoculation (12, 32, 34).

**Fig 2:**
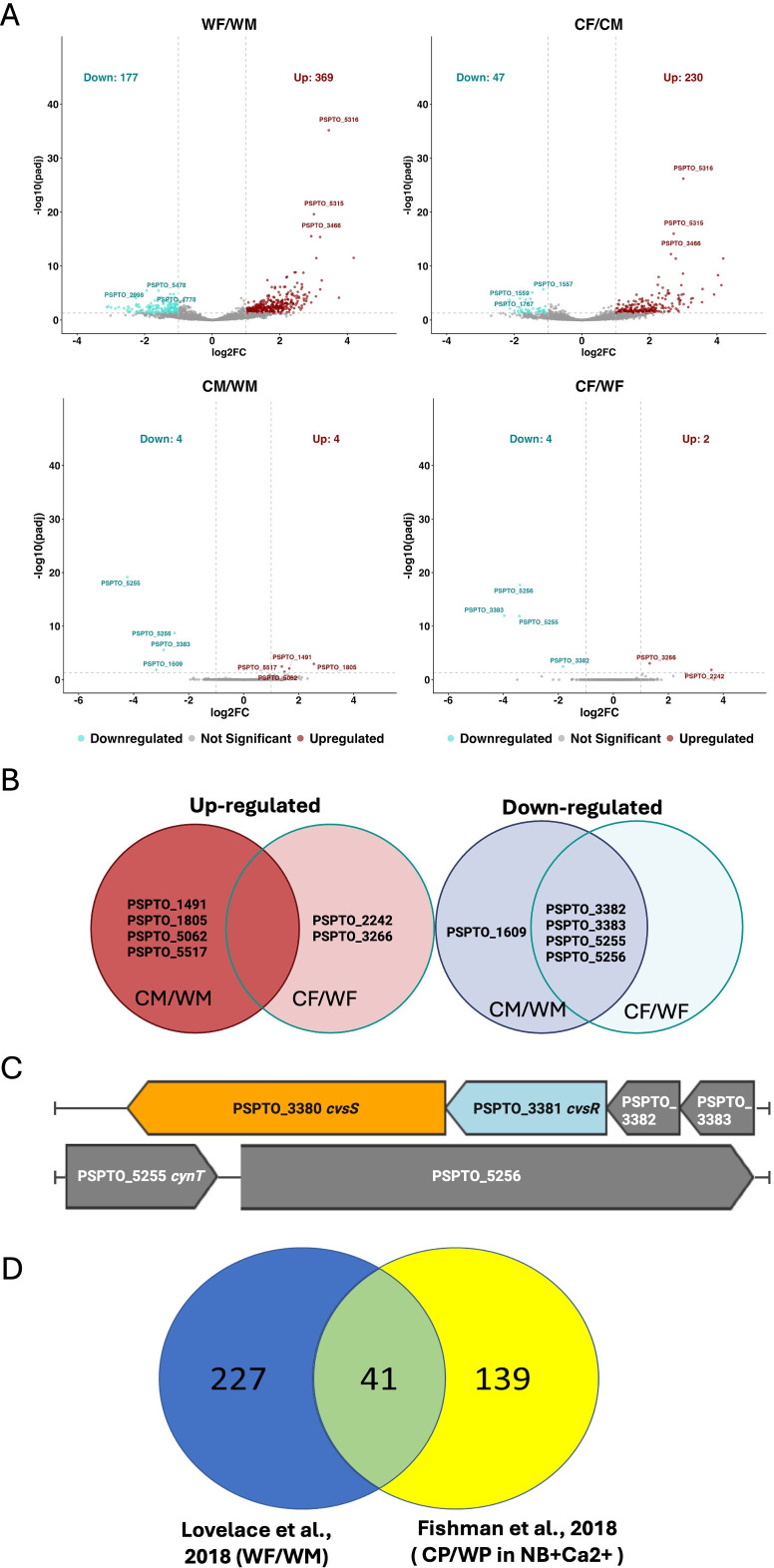
Comparison of *Pseudomonas syringae* pv. *tomato* DC3000 transcriptomes from mock (M) or flg22 (F) treatments in wild-type (W) or Δ*cvsR* (C) strains. (**A**) Volcano plots display statistical significance (–log_10_ adjusted *P* value) versus expression fold change (log_2_-transformed read counts). Red and blue points indicate significantly up- and downregulated differentially expressed genes (DEGs). Top DEGs were highlighted. (**B**) Venn diagram showing comparisons of up- (red) and downregulated (blue) DEGs between Δ*cvsR* and wild-type strains under both mock and flg22 treatments (**C**) Direct regulon of CVSR of two gene clusters. (**D**) The overlap between transcriptomic DEGs from Lovelace et al. (32) (blue; WF/WM, flg22 vs mock at five Hpi) and those from Fishman et al. (29) (yellow; CP/WP, Δ*cvsR* vs wild-type under calcium-inducing *in vitro* conditions)

The lack of pronounced transcriptomic shifts in the *cvsR* mutant, even under *in vitro* conditions, contrasts with the regulatory model proposed by Fishman et al. (29). However, variations in experimental conditions between the two studies may contribute to the difference. In the prior study, *cvsR* expression was induced by supplementation of nutrient broth with 5 mM calcium, whereas our analysis was performed under standard KB medium and *in planta* conditions. These differences in medium composition, particularly the lack of supplemental calcium, may account for the observed differences. Notably, *cvsR* expression was comparable between *in planta* conditions and cultures grown in King’s B (KB) medium (Fig. S2). In the leaf apoplast, *cvsR* mutation did not markedly alter the overall bacterial transcriptome despites the immune states of plant (Fig. 2A). The expression level of *cvsR* itself remained insignificant between mock and PTI tissue (Fig. S3). This observation supports that the host immune state remains the major determinant of bacterial gene expression during early stages of infection.

Due to the large quantity of samples collected for RNA isolation, completion of the pre-treatment and bacterial inoculation procedures required approximately 3–4 h. Accordingly, flg22 and mock pre-treatments were initiated 1 day prior to bacterial inoculation, resulting in an effective pre-treatment window ranging from 16 to 20 h depending on sampling order. Bacterial inoculation was subsequently performed in the same sequential order for each individual plant. To evaluate whether variation in pre-treatment duration influenced bacterial infection outcomes or affected virulence comparisons between wild-type and mutant strains, bacterial populations were assessed from leaves receiving the earliest (16 h) and latest (20 h) pre-treatment intervals. Although bacterial loads were slightly higher at 5 hpi in leaves receiving the 16-h flg22 pre-treatment, no significant difference was observed at 24 hpi (Fig. S1A). Both 16 h and 20 h flg22 pre-treatments conferred protection against bacterial infection, as leaves remained intact at 24 hpi compared with the symptomatic leaves observed in mock-treated controls (WT-mock). Similarly, both 16- and 20-h flg22 pre-treated leaves remained intact at 72 hpi, whereas severe tissue wilting was observed in mock-treated leaves (Fig. S1C). No significant differences in bacterial populations were detected between wild-type and Δ*cvsR* strains at either 5 or 24 hpi under mock- or flg22-pre-treated conditions (Fig. S1B). Likewise, both strains caused similar disease symptoms in mock-treated leaves at 24 and 72 hpi (Fig. S1C). Although precise control of pre-treatment duration was challenging due to the scale of sample collection, these results indicate that variation in pre-treatment timing within the tested 16–20 h window did not measurably affect bacterial populations at 24 hpi or disease symptom development relative to mock treatment. Previous work by Fishman et al. (29) reported that deletion of *cvsR* reduced disease symptoms at 4 dpi when using a lower inoculum dose (3 × 10^4^ CFU/mL). However, in our study, both strains exhibited similar phenotypes when inoculated at the substantially higher bacterial concentration (1 × 10^9^ CFU/mL) required for RNA isolation.

### The *cvsR* mutant has a reduced PTI stimulon response

To assess the role of CvsR in the bacterial PTI stimulon, we compared transcriptomic changes between wild-type “W” and Δ*cvsR* “C” strains under mock “M,” flg22-pretreated “F,” and KB medium “P” conditions. In wild-type *Pto* DC3000, the PTI stimulon (WF vs WM) triggered extensive transcriptional reprogramming, with 546 differentially expressed genes (DEGs; |log2FC| > 1, padj < 0.05), including 369 upregulated and 177 downregulated genes (Fig. 2A; Table S2.1). By contrast, the Δ*cvsR* mutant (CF vs CM) exhibited an attenuated PTI stimulon response, with only 277 DEGs (230 upregulated, 47 downregulated) (Fig. 2A; Table S2.2). The direct CvsR regulon, as previously determined based on the ChIP-seq analysis, revealed only very limited direct targets with the expression of CvsR *in vitro* (29). In naïve conditions (CM vs WM), Δ*cvsR* displayed just eight DEGs (four upregulated, four downregulated) (Fig. 2A; Table S2.3), whereas under PTI (CF vs WF), only six DEGs were identified (two upregulated, four downregulated) (Fig. 2A; Table S2.4). Venn diagram comparisons highlighted distinct sets of *cvsR*-dependent genes under naïve “M” versus PTI “F” conditions. Specifically, four genes (PSPTO_1491, PSPTO_1805, PSPTO_5062, and PSPTO_5517) were uniquely upregulated in the *cvsR* mutant under mock conditions, whereas two genes (PSPTO_2242 and PSPTO_3266) were specifically upregulated under PTI conditions (Fig. 2B). Conversely, two downregulated clusters (PSPTO_3382–3383 and PSPTO_5255–5256) were shared between naïve and PTI conditions, suggesting stable CvsR-dependent repression in both naïve and PTI tissue.

Among these, two directly regulated gene clusters—PSPTO_3382–3383 and PSPTO_5255–5256—were previously identified as direct CvsR targets via i*n vitro* ChIP-seq experiments (29). Our data confirmed that these genes represent the core *cvsR* regulon. Gene schematic analysis showed that CvsS/CvsR directlyregulate a putative operon containing the β-carbonic anhydrase (*cynT*; PSPTO_5255) and an adjacent major facilitator superfamily (MFS) transporter PSPTO_5256 (Fig. 2C). Raw reads from RNA-seq indicated the depleted transcripts of both *cvsS*/*cvsR* and PSPTO_5255-5256 in Δ*cvsR* (Fig. S3). Notably, the co-transcribed MFS transporter with the carbonic anhydrases was hypothesized to function in bicarbonate or carbonic acid transport to prevent surface-associated calcium phosphate precipitation and promote swarming ability (37).

Despite the largely unchanged global transcriptome pattern between wild-type and Δ*cvsR* strains *in planta*, the reduced number of differentially expressed genes under PTI conditions (CF/CM) suggests that CvsR minorly contributes to the bacterial PTI stimulon response. This finding indicates that while *cvsR* deletion does not broadly alter transcriptional profiles, but rather likely modulates specific transcriptional responses important for adaptation to the host immune environment indirectly. Compared with the cvsR-dependent differentially expressed genes (DEGs) under calcium-induced *in vitro* conditions (29), only two CvsR directly-regulated clusters remained responsive *in planta*. These results also imply that *cvsR* and its direct regulon may play a more pronounced role in bacterial adaptation to environmental cues outside the plant apoplast. *In planta*, the CvsS/CvsR system appears to primarily control a core operon comprising *cynT* and its adjacent transporter. This operon represents the only consistently regulated CvsR-dependent locus detected *in planta* other than the *cvsS/R* cluster itself, supporting its designation as the core *cvsR* regulon during host interaction. Given the hypothesized role of these proteins in bicarbonate or carbonic acid transport (37), it would be informative to examine whether the global transcriptome profiles differ between *cvsR* and *cynT* mutants to further elucidate the functional significance of this regulatory module in bacterial adaptation to the host apoplast.

### CvsR-dependent modulation of PTI-associated pathways

**Fig 3:**
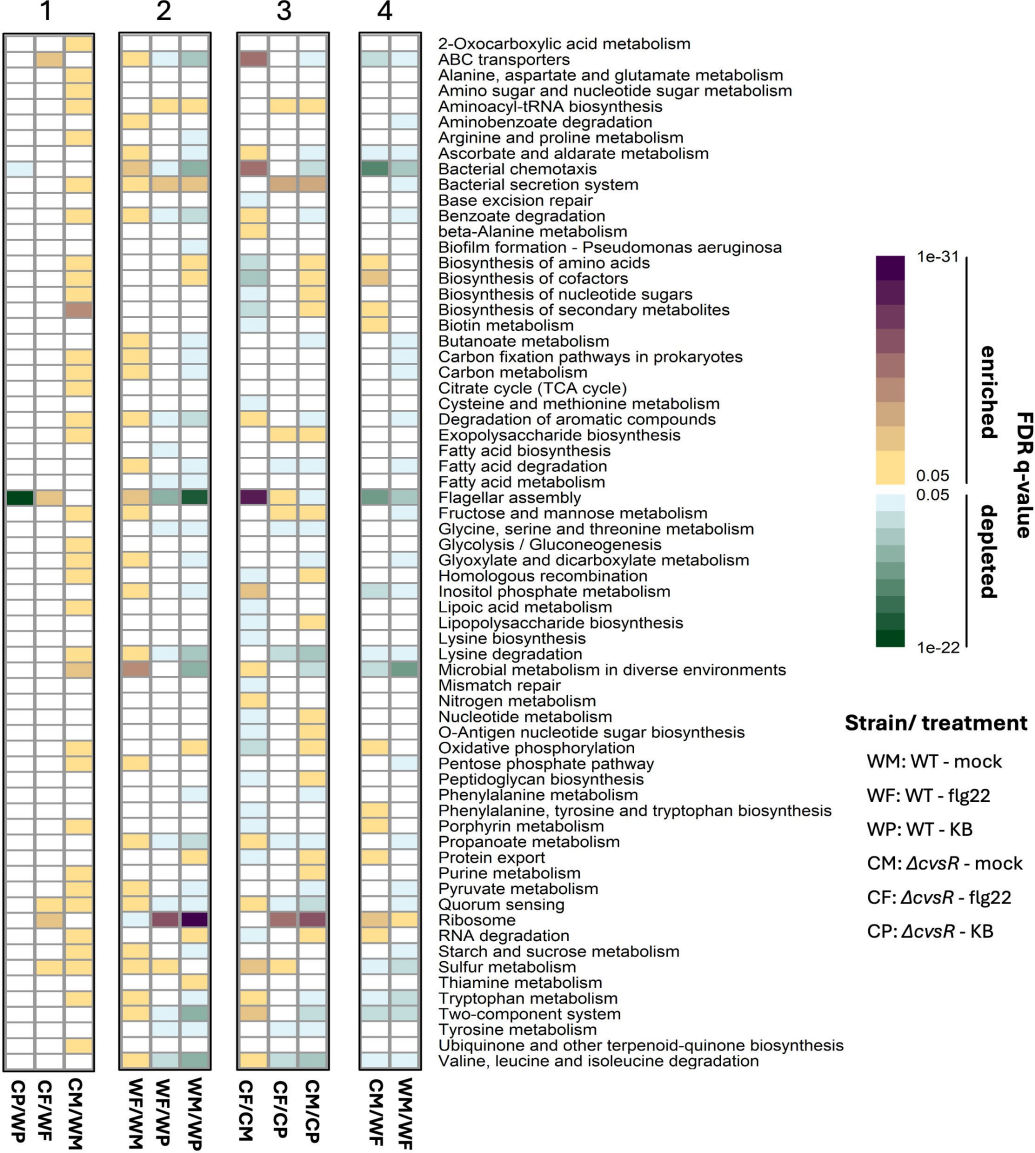
Differentially expressed KEGG (Kyoto Encyclopedia of Genes and Genomes) pathways for various comparisons in *Pseudomonas syringae* pv. *tomato* DC3000. Heat map of q-values for KEGG pathways representing the differentially expressed genes in different comparisons of Δ*cvsR* (C), wild type (W), flg22 treatment (F), and mock control (M). Differentially expressed pathways were identified using the goseq function in the gage v.2.24.0 R package. Color scale corresponds to the FDR-adjusted q-values, with darker shades indicating higher statistical significance. Pathways are classified as upregulated (purple scale) or downregulated (green scale) based on differential gene expression patterns.

To gain a systems-level view of the CvsR contribution to the PTI stimulon, we conducted KEGG pathway enrichment analyses across all experimental groups (Fig. 3). The heatmap illustrates the strength of statistical confidence (–log₁₀ q-value) for each pathway rather than absolute gene expression levels, thereby emphasizing the robustness of enrichment rather than the magnitude of transcriptional change or the number of genes in individual pathways. Comparison of Δ*cvsR* and wild-type strains under *in vitro* conditions (CP/WP) revealed only two significantly depleted pathways—bacterial chemotaxis and flagellar assembly—indicating reduced expression of motility-related genes in the absence of CvsR. Notably, CvsR-dependent regulation of motility-associated pathways was not observed *in planta*, under either naïve (CM/WM) or PTI (CF/WF) conditions, suggesting that the suppression of bacterial motility genes *in planta* overrides the regulatory effect by CvsR under *in vitro* conditions. The second block of the heatmap highlighted KEGG pathways enriched in wild-type *Pto* DC3000 during PTI (WF/WM). Consistent with previous transcriptomic studies, PTI led to significant enrichment of metabolic and stress-response pathways, reflecting broad transcriptional reprogramming characteristic of the PTI stimulon. The third block summarized CvsR-dependent pathways under host-associated conditions. While the impact of CvsR was modest in naïve tissue (CM/WM), a stronger effect was observed during PTI (CF/WF), where pathway enrichment patterns were generally attenuated in the mutant relative to the wild type. This indicates that CvsR amplifies the magnitude and breadth of the PTI-responsive transcriptional network *in planta*.

The *in vitro* study by Fishman et al. (29) identified CvsR-dependent transcriptome expression profiles under calcium-augmented nutrient broth. The differentially expressed genes (DEGs) we observed (32) shared approximately 16% overlap with the PTI stimulon and 22% with the CvsR *in vitro* regulon (Fig. 2D; Table S3). Based on this similarity, we hypothesized that the PTI may suppress CvsR signaling. Thus, a *cvsR* mutant would have transcriptional responses mimicking those typically observed in response to the PTI stimulon. In the fourth block, we examined the Δ*cvsR* mutant in naïve tissue relative to the wild-type strain in PTI-activated tissue (CM/WF). If the Δ*cvsR* mutant showed PTI stimulon-like expression patterns under mock conditions, we would expect reduced transcriptional differences between Δ*cvsR* mock and wild-type flg22 conditions. However, a large number of significantly enriched pathways were still observed in the (CM/WF) comparison, and the expression pattern did not resemble that of the PTI regulon (WM/WF). This suggested that the regulatory network of CvsR makes only limited contributions to the bacterial response to PTI.

### CvsR modulation of bacterial secretion systems

**Fig 4:**
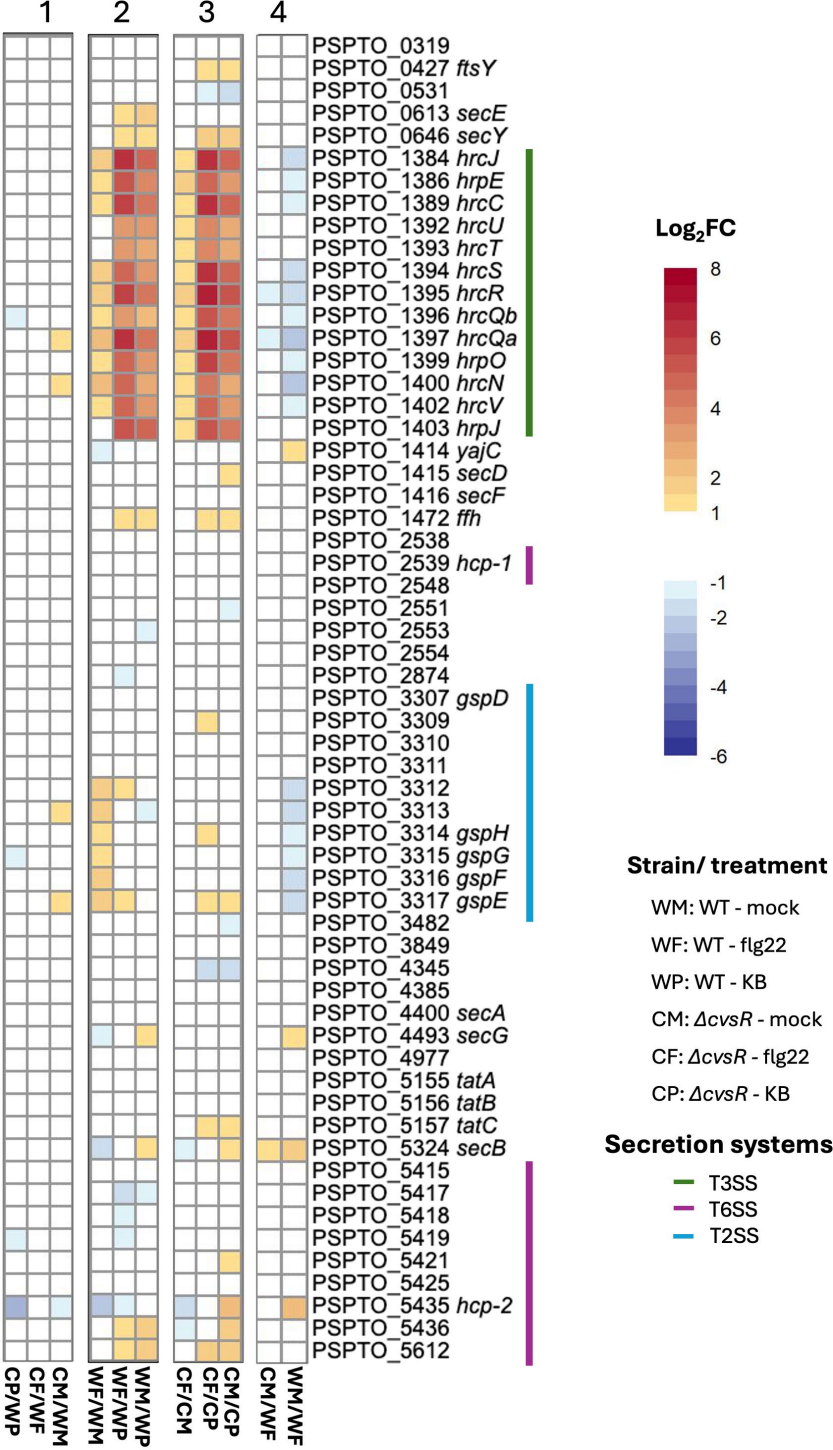
Gene expression level of bacterial secretion system genes. Heat map displays the log_2_ fold change (log_2_FC) in mean normalized counts for *Pseudomonas syringae* pv. *tomato* DC3000 bacterial secretion genes from the KEGG in different comparisons of Δ*cvsR* (C), wild type (W), flg22 treatment (F), and mock control (M). Colored cells indicate significant differential expression: red corresponds to upregulation and blue to downregulation (adjusted *P* value < 0.05); white denotes genes not significantly changed (adjusted *P* value > 0.05).

In our study, the type III secretion system (T3SS) genes are slightly upregulated during pattern-triggered immunity (PTI) as compared with naïve plant infections (Fig. 4) (WF/WM, and CF/CM). This result diverges somewhat from the findings of Lovelace et al. (32), where PTI induction prior to infection was associated with mildly reduced T3SS gene expression at 5 h post-inoculation but is consistent with patterns observed in Smith et al. (38), where T3SS expression is maintained at a higher level in PTI tissue than naïve tissue at later time points (24 h post-inoculation).

There was an increased induction of T3SS genes in a *cvsR* mutant during PTI relative to the WT strain (WF/WM vs CF/CM). This suggested CvsR involved in suppression of these genes and contradict to the previous reported phenotype in reduced virulence of mutants (39). Paradoxically, *in vitro* basal expression of T3SS genes was lower in Δ*cvsR* than that in wild type (Fig. 4, block1, CP/WP), aligning with the *in vitro* setting from previous report by Fishman et al. (29). However, the reduction in T3SS genes in Δ*cvsR* was not observed *in planta* (Fig. 4, block 1, CF/WF; CM/WM). The expression of type II secretion system (T2SS) pathway genes remained largely unchanged in wild-type strains during *in planta* colonization, but in the absence of *cvsR*, T2SS gene are upregulated (Fig. 4, block 3).

Many plant-associated bacteria that possess the T6SS have strong competitive fitness when cultured with other bacteria, with few examples suggested T6SS contributed to the pathogen virulence (40, 41). In the PTI tissue, most genes associated with T6SS, including *hcp-2*, were downregulated in wild-type strain (Fig. 4, block2, WF/WM, WF/WP). Mutation of *cvsR* diminishes the degree of T6SS downregulation and was even elevated in naïve condition compared with *in vitro* culture (Fig. 4, block 3, CF/CP, CM/CP). Without *cvsR*, the PTI-induced suppression is lost, resulting in relatively sustained or less reduced levels of T6SS gene expression particularly in immune-challenged host tissue.

### CvsR modulation of bacterial sulfate and sulfonate importers and sulfur metabolism genes

**Fig 5:**
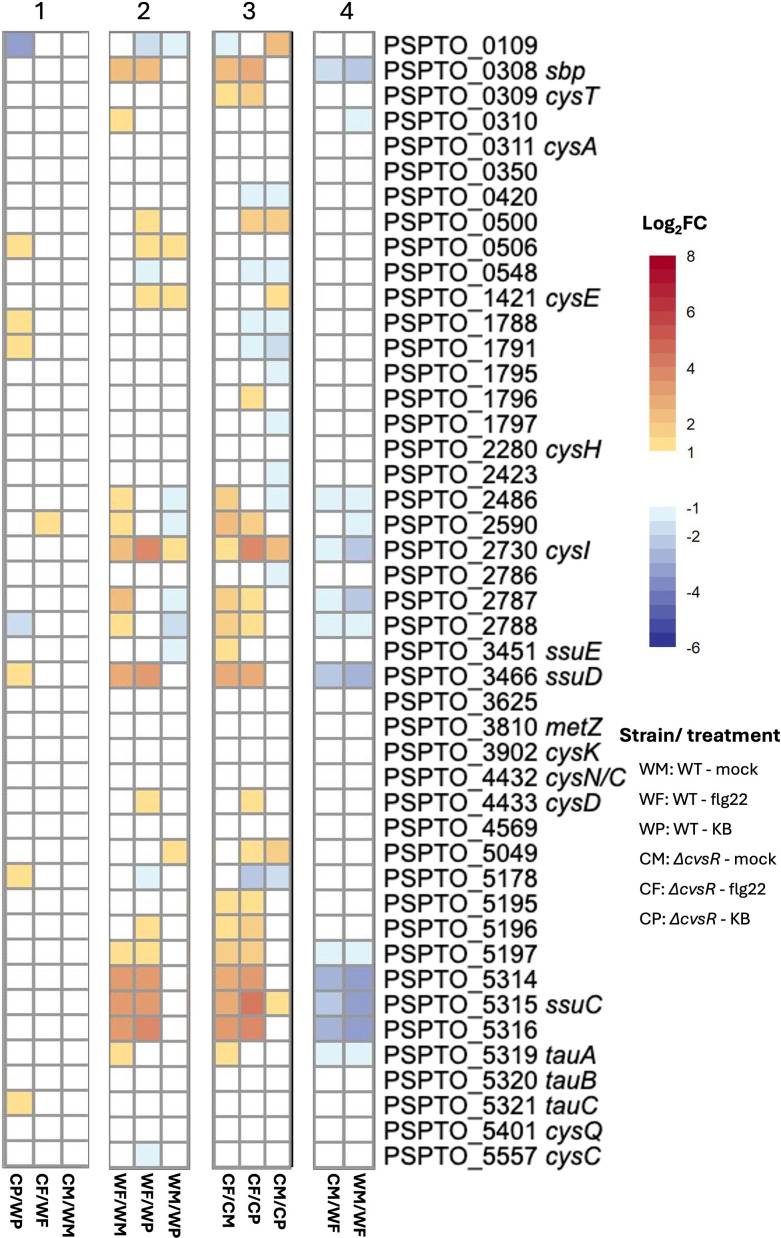
Gene expression level of sulfur metabolism genes. Heat map displays the log_2_ fold change (log_2_FC) in mean normalized counts for *Pseudomonas syringae* pv. *tomato* DC3000 sulfur metabolism genes from the KEGG in different comparisons of Δ*cvsR* (C), wild type (W), flg22 treatment (F), and mock control (M). Colored cells indicate significant differential expression: red corresponds to upregulation and blue to downregulation (adjusted *P* value < 0.05); white denotes genes not significantly changed (adjusted *P* value > 0.05).

The CvsS/CvsR two component system were previously observed to contribute to regulation of sulfate and sulfonate importers and sulfur metabolism (29). While sulfur metabolism did not show significant changes at the pathway level in the *in vitro* comparison (Fig. 3, CP/WP), transcriptome data revealed modest downregulation of individual sulfur metabolism–associated genes (e.g., *PSPTO_0109*, *FAD-dependent oxidoreductase*) in the Δ*cvsR* under *in vitro* conditions (Fig. 5, block 1, CP/WP). However, this effect does not extend to the genes for sulfate and sulfonate periplasmic biding proteins *sbp* and *sfbp* (PSPTO_5316), contrasting with prior *in vitro* study by calcium augmentation (29). *In planta*, this repression largely disappears, as indicated by negligible changes in log_2_ fold-change for gene expression across the entire sulfur metabolism pathway. The only notable exception is PSPTO_2590, which encodes a dimethylsulfone monooxygenase *sfnG*. Strong induction of genes involved in sulfate import, including *sbp* and *sfbp*, was detected during plant infection, with this upregulation being even higher in PTI-induced tissue, consistent with previous findings by Lovelace et al. (32). The loss of *cvsR* further amplifies this response (Fig. S7).

Sulfate metabolism genes are among the most distinct DEGs during bacterial responses to PTI (32). Our *in vitro* data showed no major reduction in the expression of the sulfate metabolism pathway (KEGG) in King’s B media (CP/WP), although several genes exhibited mild downregulation, consistent with the *in vitro* study by Fishman et al. (29). However, this reduction was minor compared with the calcium-induced repression reported previously. Moreover, we did not observe strong suppression of *sbp* and *sfbp*, which were the least affected sulfate metabolism genes in the *cvsR* mutant. Sulfate metabolism genes exhibited higher expression under PTI stimulation (Fig. 5, WF/WM, WF/WP, CF/CM, CF/CP; Fig. S4), which likely explains the stronger induction observed in the *cvsR* mutant *in planta* compared with *in vitro*, reflecting *cvsR*-dependent downregulation of this pathway under *in vitro* conditions.

### CvsR modulation of bacterial chemotaxis and flagellar regulation

**Fig 6:**
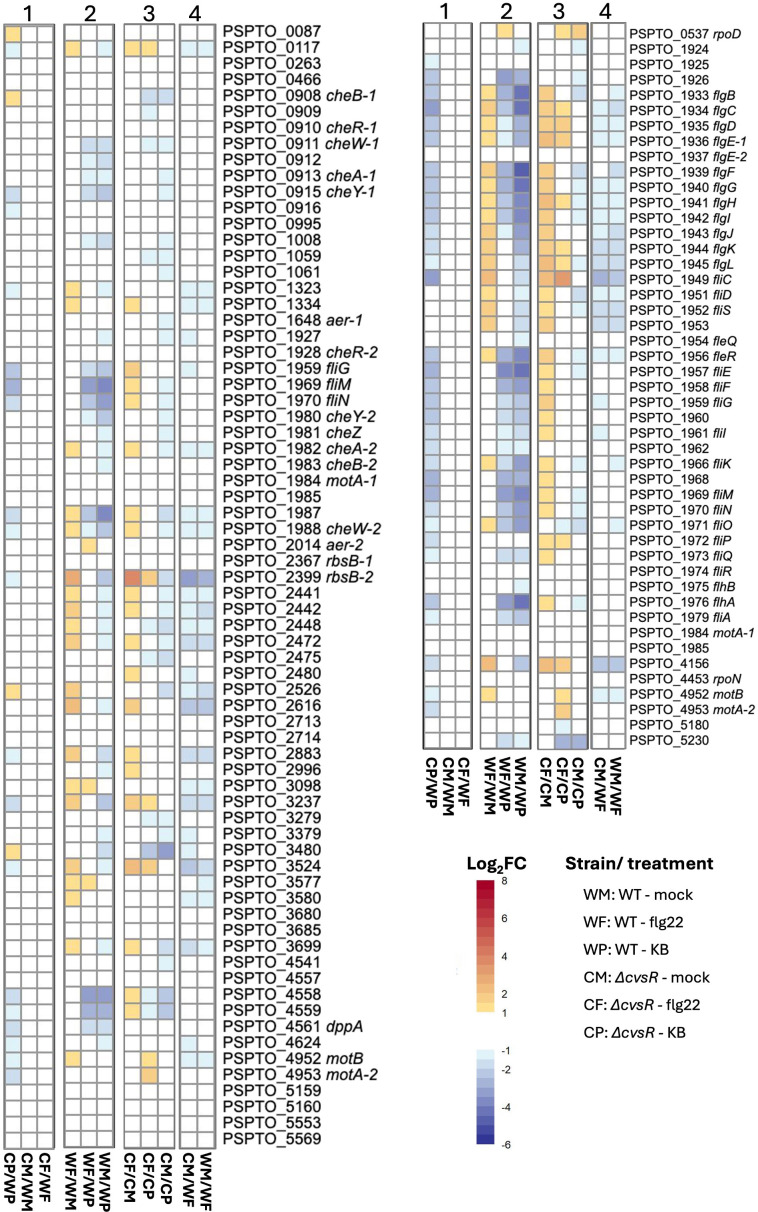
Gene expression level of motility genes. Heat map displays the log_2_ fold change (log_2_FC) in mean normalized counts for *Pseudomonas syringae* pv. *tomato* DC3000 sulfur metabolism genes from the KEGG in different comparisons of Δ*cvsR* (C), wild type (W), flg22 treatment (F), and mock control (M). Colored cells indicate significant differential expression: red corresponds to upregulation and blue to downregulation (adjusted *P* value < 0.05); white denotes genes not significantly changed (adjusted *P* value > 0.05).

Distinctive patterns of bacterial chemotaxis and flagella assembly genes under *in vitro* and *in planta* conditions provided evidence of limited impact of *cvsR* during plant infection (Fig. 6; Fig. S5 and S6). Under *in vitro* condition, chemotaxis and flagella pathways were downregulated in the Δ*cvsR* compared with the wild type (Fig. 6, block1, CP/WP), consistent with the impaired motility observed in Δ*cvsR*. This finding corroborates previous reports demonstrating a cvsR-dependent swarming phenotype and defect in motility upon *cvsR* disruption (29, 37). However, when these genes are examined *in planta*, regardless of immune status (Fig. 6, block 1, CM/WM and CF/WF), chemotaxis and motility gene expression do not significantly differ between Δ*cvsR* and wild type. Surprisingly, while flagella and chemotaxis genes are much less expressed *in planta* than *in vitro* in the wild-type strains (Fig. 6, block 2), the expression of many genes (e.g., *motA-2*, *mot-B*, *fliC*) displayed upregulation *in planta* than *in vitro* with the mutation of *cvsR* with higher expression level in PTI compared with naïve tissue (Fig. 6, block 3).

In contrast to the inducing conditions under which 199 genes were reported as potential direct targets of CvsR (29), our data suggest that CvsR exhibits a highly restricted core regulon in planta. This regulon appears to be confined to autoregulation of the cvsS/cvsR two-component system and regulation of cynT (carbonic anhydrase) and its associated transporter PSPTO_5256, with no evidence for direct regulation of additional transcription factors. Thus, the subtle CvsR modulation of multiple pathways including motility and sulfur metabolism may be the results of altered expression of carbonic anhydrase expression. Carbonic anhydrase may reduce local pH on the surface of cells through production of carbonic acid (37). Thus, the reduced *cynT* expression in Δ*cvsR* may result in increased local pH around the colonization zone in the apoplast. During PTI, the apoplast undergoes a significant pH increase (17). Thus, the observed partial overlap between a *cvsR*-modulated genes and the response to the PTI stimulon may be attributable to bacterial responses to increased local pH.

### Conclusion

**Fig 7:**
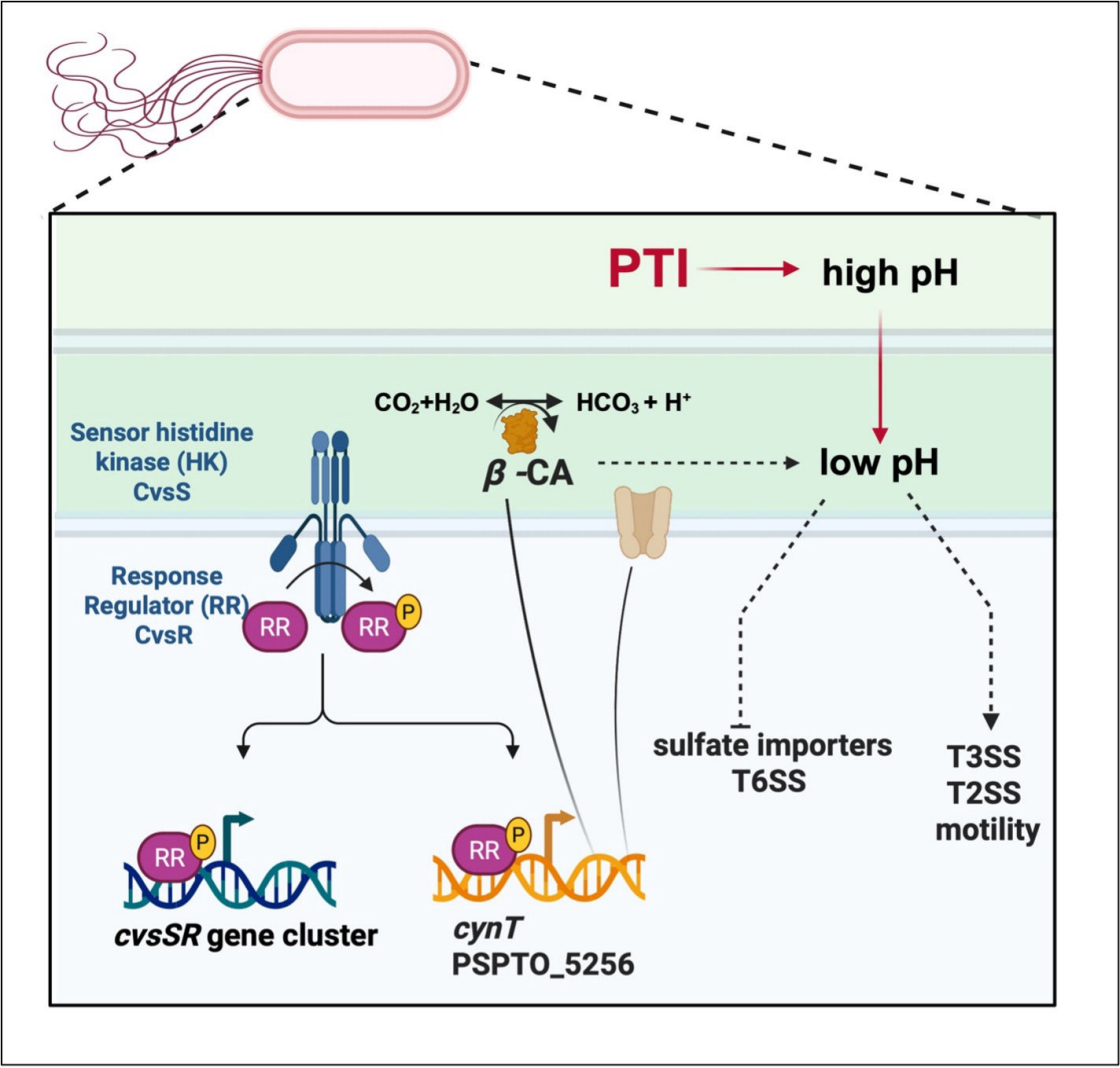
Schematic model of function of CvsR of *Pseudomonas syringae* pv. *tomato* DC3000 in *Arabidopsis* during pattern-triggered immunity (PTI). Solid arrows represent known activation. Blunt arrows represent known inhibition. Dashed arrows represent hypothetical interactions. Created with BioRender.com

In this study, we established an optimized *in planta* bacterial transcriptome profiling approach that achieves exceptionally high bacterial read recovery through combined host rRNA depletion and physical enrichment. This method enables reliable resolution of bacterial transcriptional dynamics under immunity-activated states. Our results demonstrate that while CvsR contributes minimally to global transcriptional reprogramming within the host, it retains distinct regulatory activity over a small, conserved regulon, primarily encompassing the β-carbonic anhydrase (PSPTO_5255) and its adjacent MFS transporter (PSPTO_5256) *in planta* (Fig. 7). Under *in vitro* conditions, CvsR regulates motility- and sulfur metabolism-associated pathways, consistent with previous reports, but these effects are largely attenuated *in planta*. The attenuated PTI stimulon responses observed in the Δ*cvsR* mutant further suggests that CvsR fine-tunes bacterial responses to immune-induced environmental cues rather than acting as a major transcriptional driver. Collectively, these findings indicate that CvsR functions as a context-dependent regulator with limited influence in the plant apoplast, yet its role may be more pronounced in mediating bacterial adaptation to external or pre-infection environments. Future characterization of the cognate histidine kinase CvsS activation, through the use of hybrid reporter systems (42), for example, can be useful for pinpointing the PTI-induced ligand perceived by this TCS and for elucidating the mechanistic basis of CvsSR signaling during host immune activation.

## MATERIALS AND METHODS

### Plant tissue preparation and growth condition

*Arabidopsis thaliana* (Col-0) seeds were sown under the mesh-covered pot, as described previously (32). Plants were grown in a growth chamber (Conviron A1000) for 4.5 weeks under long-day conditions at 23°C (14-h day and 10-h night) at 70-μmol light settings. One day prior to treatment, plants were removed from the growth chamber and were placed in a growth room under 12-h day and 12-h night conditions. Then, 3 to 5 fully expanded leaves on each plant on each plant were infiltrated with either 1 μM flg22 in 0.1% DMSO or with 0.1% DMSO by 1-mL blunt syringes on the abaxial surface 1 day prior to the bacterial inoculation. The pre-treatments ranged from 16 to 20 h prior to bacterial inoculation due to the large quantity of sample collection.

### Bacterial inoculation

*Pseudomonas syringae* pv. *tomato* DC3000 (*Pto* DC3000) was prepared as lawn, growing on King’s B (KB) (43) agar supplemented with 40 μg/mL rifampicin and was grown overnight at 28°C. Inoculum was harvested from plates by washing out bacterial smear from the plate surface and suspended and washed twice in 0.25 mM MgCl_2_.OD_600_ of 0.8 (1 × 10^9^ CFU/mL) was reached as the final concentration for inoculum. Bacterial inoculum was infiltrated with a blunt-end syringe into treated leaves as described above. Plants were allowed to dry at ambient temperature (22°C) on bench for 5 h before sampling for RNA isolation.

### Extraction of bacteria from infected plants

At 5 hpi, leaves from each treatment were harvested by cutting at the leaf blade junction. Isolation of bacteria from leaf apoplast was followed from the previous method (32) with modification. Briefly, 80–100 leaves were arranged on four sheets of parafilm, rolled and inserted into the barrels of four 20-mL syringes. An ice-cold RNA stabilizing buffer (Invitrogen) was poured into each syringe, which was sealed and vacuum infiltrated at 95 kPa for 2 min, followed by a slow release of the vacuum. The RNA stabilizing buffer was spun out from apoplast at 1,000 × *g* for 10 min at 4°C to isolate bacterial RNA.

The flow-through was pooled for each biological replicate from each treatment and was concentrated by syringe filtration, using a 0.20-μm Micropore membrane (Millipore). Filters were placed in homogenization tubes, were flash frozen in liquid nitrogen, and were stored at −80°C for RNA extraction.

### Bacterial RNA isolation and sequencing

The filter membranes were homogenized in Trizol reagent (Thermo Fisher Scientific, Waltham, MA) for 1 min at 1,750 Hz, using a Geno/Grinder (Thermo Fisher Scientific, Waltham, MA) with 3 × 3 mm high density zirconium beads followed by the chloroform-ethanol isolation from manufacturer (TRIzol Reagent User Guide, Pub. No. MAN0001271 D). For *in vitro* samples, 1 mL of the 1 × 10^9^ CFU/mL bacterial inoculum in 0.25 mM MgCl_2_ was pelleted, and RNA was extracted followed by the same procedure above.

RNA samples were additionally treated with TURBO DNase (Invitrogen, Carlsbad, CA) to eliminate genomic DNA contamination. RNA was quantified using the NanoDrop OneC Microvolume Spectrophotometer (Thermo Fisher Scientific, Waltham, MA). Three independent bacterial suspensions started from three colonies were sampled for a total of three biological replicates. The plant samples were depleted of rRNA using the QIAseq FastSelect –rRNA Plant Kit (Qiagen, Germantown, MD), and bacteria samples were depleted of rRNA using the QIAGEN FastSelect rRNA HMR Kit (Qiagen, Germantown, MD). Next, RNA sequencing libraries were constructed with the NEBNext Ultra II RNA Library Preparation Kit for Illumina by following the manufacturer’s recommendations (New England Biolabs, lpswich, MA). Paired-end 150-nt reads were sequenced using the Nova-seq platform (Illumina, San Diego, CA).

### RNA-Seq data analysis

Reads were quality trimmed using Trimmomatic (39) and were aligned to the RefSeq *P. syringae* pv. *tomato* DC3000 genome (NC_004278.1, NC_004632.1, NC_004633.1), using Bowtie2 (44). Counts of RNA-Seq fragments were computed for each annotated gene, from reads per kilobase million values, using the StringTie script (45). DEGs were identified from gene counts for each sample, using the Bioconductor package DESeq2 version 3.21 (46). DEGs were selected based on log2-transformed and normalized mean counts that have an adjusted *P* value below a false discovery rate (FDR) cutoff of 0.05. DEGs set from each comparison were extracted with an additional cutoff of |log2FC| > 1.0.

Principal components analysis of log2-transformed normalized counts was performed for all treatments and replicates, using the rlog function in DESeq2. Volcano plot was performed on log2 fold changes in mean normalized counts between treatments using DESeq2, and heatmaps were generated using the R package pheatmap version 1.0.13.

KEGG analysis was conducted using log2 fold change values of normalized mean counts for annotated genes using the Bioconductor package, gage version 2.24.0 (47). Gene sets of metabolic pathways were obtained from the KEGG pathway database, using the organismal code “pst” for *Pto* DC3000. Significant gene sets were identified from log2 fold changes between treatments at similar time points and were selected based on an FDR q value cutoff of 0.05. The data represented enrichment of pathways rather than the expression level between the treatment pair.

### Bacterial load quantification and symptom

Four replicates of 4.5-week-old *A. thaliana* Col-0 plants were treated with 1 μM flg22 in 0.1% DMSO or 0.1% DMSO 16 or 20 h prior to inoculation, as described above. Bacterial quantification was performed at 5/24 h post‐infection (hpi). Four 4‐mm‐diameter leaf discs (∼0.5 cm^2^ total area) per treatment were collected using a biopsy punch. Tissues were homogenized in 0.1 mL of 0.25 mM MgCl_2_ for 2 min using a SpeedMill Plus homogenizer (Analytik Jena, Jena, Germany), with subsequent bacterial enumeration via serial dilution spot plating. For each genotype and treatment, four plants were used per replicate, and the experiment was independently repeated twice (*n* = 8). Bacterial load was expressed as mean log_10_ (CFU cm^−2^) ± standard error (SE). Statistical comparisons between mutants and Col‐0 wild type were performed using a paired two‐tailed Student’s *t*‐test, with significance defined as *P* < 0.05. Symptom of leaves was assessed at 24/72 hpi. qPCR analysis on selected genes of interest

Four replicates of 4.5-week-old *A. thaliana* Col-0 plants were treated with 1 μM flg22 in 0.1% DMSO or 0.1% DMSO 16 h prior to inoculation, as described above. The bacterial inoculum of *Pto* DC3000 wild type or *cvsR* mutant was prepared as described above and was further diluted to a final concentration of approximately 1 × 10^9^ CFU/mL and was infiltrated into marked leaves of all plants. At 5 hpi, inoculated leaves from a single plant were harvested and homogenized. Ground tissues were set for RNA extraction using TriZol and TURBO-DNase cleanup, as described above. Four independent samples of pelleted initial inoculum were also flash-frozen in liquid nitrogen for RT-qPCR analysis. cDNA synthesis, RT-qPCR, and normalization were conducted based on previously described procedures (38).

Normalized cDNA was tested from four treatments, i.e., wild-type/*cvsR* mutant strain from KB, naïve host tissue, and PTI tissue. These samples were tested for relative expression of two genes of interest, *sbp* and *sfbp*. Samples were grouped by biological replicate set and were tested against five genes of interest, two previously validated, *recA* as a reference gene, inoculum, and *Pto* DC3000-specific 16S rRNA within the same plate (38). Relative expression of *in planta* samples normalized to the inoculum sample, measured as the NRQ of a gene of interest, was calculated as described previously (38). Relative expression of bacteria exposed to PTI relative to bacteria during naïve host infection, measured as the NRQ of a gene of interest, was calculated similarly to that above; a Student’s *t*-test was performed on log2 NRQ values for each gene of interest against the wild-type sample in naïve tissue (WM).

## Data Availability

The RNA-seq data used in this study are deposited in the National Center for Biotechnology Information Gene Expression Omnibus database with accession number: GSE327564.

## SUPPLEMENTAL MATERIAL

Supplemental figures spectrum.04124-25-s0001.docx Fig. S1 to S7.

Supplemental tables spectrum.04124-25-s0002.xlsx Tables S1 to S3.

ASM does not own the copyrights to Supplemental Material that may be linked to, or accessed through, an article. The authors have granted ASM a non-exclusive, world-wide license to publish the Supplemental Material files. Please contact the corresponding author directly for reuse.

## References

[1] Jones JDG, Dangl JL. 2006. The plant immune system. Nature 444:323–329. doi:10.1038/nature05286 17108957

[2] Ngou BPM, Jones JDG, Ding P. 2022. Plant immune networks. Trends Plant Sci 27:255–273. doi:10.1016/j.tplants.2021.08.012 34548213

[3] Gómez-Gómez L, Bauer Z, Boller T. 2001. Both the extracellular leucine-rich repeat domain and the kinase activity of FLS2 are required for flagellin binding and signaling in *Arabidopsis*. Plant Cell 13:1155–1163. doi:10.1105/tpc.13.5.1155 11340188 PMC135565

[4] Chinchilla D, Bauer Z, Regenass M, Boller T, Felix G. 2006. The *Arabidopsis* receptor kinase FLS2 binds flg22 and determines the specificity of flagellin perception. Plant Cell 18:465–476. doi:10.1105/tpc.105.036574 16377758 PMC1356552

[5] Kunze G, Zipfel C, Robatzek S, Niehaus K, Boller T, Felix G. 2004. The N terminus of bacterial elongation factor Tu elicits innate immunity in *Arabidopsis* plants. Plant Cell 16:3496–3507. doi:10.1105/tpc.104.026765 15548740 PMC535888

[6] Felix G, Boller T. 2003. Molecular sensing of bacteria in plants. The highly conserved RNA-binding motif RNP-1 of bacterial cold shock proteins is recognized as an elicitor signal in tobacco. J Biol Chem 278:6201–6208. doi:10.1074/jbc.M209880200 12471032

[7] Yuan M, Ngou BPM, Ding P, Xin X-F. 2021. PTI-ETI crosstalk: an integrative view of plant immunity. Curr Opin Plant Biol 62:102030. doi:10.1016/j.pbi.2021.102030 33684883

[8] Zipfel C, Robatzek S, Navarro L, Oakeley EJ, Jones JDG, Felix G, Boller T. 2004. Bacterial disease resistance in *Arabidopsis* through flagellin perception. Nature 428:764–767. doi:10.1038/nature02485 15085136

[9] Crabill E, Joe A, Block A, van Rooyen JM, Alfano JR. 2010. Plant immunity directly or indirectly restricts the injection of type III effectors by the *Pseudomonas syringae* type III secretion system. Plant Physiol 154:233–244. doi:10.1104/pp.110.159723 20624999 PMC2938138

[10] Chen H-C, Newton CJ, Diaz G, Zheng Y, Kong F, Yao Y, Yang L, Kvitko BH. 2025. Proteomic snapshot of pattern triggered immunity in the *Arabidopsis* leaf apoplast. Plant J 123:e70498. doi:10.1111/tpj.70498 40991770 PMC12459878

[11] Lewis LA, Polanski K, de Torres-Zabala M, Jayaraman S, Bowden L, Moore J, Penfold CA, Jenkins DJ, Hill C, Baxter L, Kulasekaran S, Truman W, Littlejohn G, Prusinska J, Mead A, Steinbrenner J, Hickman R, Rand D, Wild DL, Ott S, Buchanan-Wollaston V, Smirnoff N, Beynon J, Denby K, Grant M. 2015. Transcriptional dynamics driving MAMP-triggered immunity and pathogen effector-mediated immunosuppression in *Arabidopsis* leaves following infection with *Pseudomonas syringae* pv *tomato* DC3000. Plant Cell 27:3038–3064. doi:10.1105/tpc.15.00471 26566919 PMC4682296

[12] Nobori T, Velásquez AC, Wu J, Kvitko BH, Kremer JM, Wang Y, He SY, Tsuda K. 2018. Transcriptome landscape of a bacterial pathogen under plant immunity. Proc Natl Acad Sci USA 115:E3055–E3064. doi:10.1073/pnas.1800529115 29531038 PMC5879711

[13] Zhou J-M, Zhang Y. 2020. Plant immunity: danger perception and signaling. Cell 181:978–989. doi:10.1016/j.cell.2020.04.028 32442407

[14] Miao P, Wang H, Wang W, Wang Z, Ke H, Cheng H, Ni J, Liang J, Yao Y-F, Wang J, Zhou J-M, Lei X. 2025. A widespread plant defense compound disarms bacterial type III injectisome assembly. Science 387:eads0377. doi:10.1126/science.ads0377 40014714

[15] Yu K, Liu Y, Tichelaar R, Savant N, Lagendijk E, van Kuijk SJL, Stringlis IA, van Dijken AJH, Pieterse CMJ, Bakker PAHM, Haney CH, Berendsen RL. 2019. Rhizosphere-associated *Pseudomonas* suppress local root immune responses by gluconic acid-mediated lowering of environmental pH. Curr Biol 29:3913–3920. doi:10.1016/j.cub.2019.09.015 31668625

[16] Dora S, Terrett OM, Sánchez-Rodríguez C. 2022. Plant-microbe interactions in the apoplast: communication at the plant cell wall. Plant Cell 34:1532–1550. doi:10.1093/plcell/koac040 35157079 PMC9048882

[17] Yang Z, Wang H, Keebler R, Lovelace A, Chen H-C, Kvitko B, Swingle B. 2024. Environmental alkalization suppresses deployment of virulence strategies in *Pseudomonas syringae* pv. *tomato* DC3000. J Bacteriol 206:e0008624. doi:10.1128/jb.00086-24 39445803 PMC11580431

[18] Whalen MC, Innes RW, Bent AF, Staskawicz BJ. 1991. Identification of *Pseudomonas syringae* pathogens of *Arabidopsis* and a bacterial locus determining avirulence on both arabidopsis and soybean. Plant Cell 3:49–59. doi:10.1105/tpc.3.1.49 1824334 PMC159978

[19] Buell CR, Joardar V, Lindeberg M, Selengut J, Paulsen IT, Gwinn ML, Dodson RJ, Deboy RT, Durkin AS, Kolonay JF, et al.. 2003. The complete genome sequence of the *Arabidopsis* and tomato pathogen *Pseudomonas syringae* pv. *tomato* DC3000. Proc Natl Acad Sci USA 100:10181–10186. doi:10.1073/pnas.1731982100 12928499 PMC193536

[20] Xin XF, Kvitko B, He SY. 2018. *Pseudomonas syringae*: what it takes to be a pathogen. Nat Rev Microbiol 16:316–328. doi:10.1038/nrmicro.2018.17 29479077 PMC5972017

[21] Guo M, Tian F, Wamboldt Y, Alfano JR. 2009. The majority of the type III effector inventory of *Pseudomonas syringae* pv. *tomato* DC3000 can suppress plant immunity. Mol Plant Microbe Interact 22:1069–1080. doi:10.1094/MPMI-22-9-1069 19656042 PMC2778199

[22] Kvitko BH, Park DH, Velásquez AC, Wei C-F, Russell AB, Martin GB, Schneider DJ, Collmer A. 2009. Deletions in the repertoire of *Pseudomonas syringae* pv. *tomato* DC3000 type III secretion effector genes reveal functional overlap among effectors. PLoS Pathog 5:e1000388. doi:10.1371/journal.ppat.1000388 19381254 PMC2663052

[23] Lee SE, Gupta R, Jayaramaiah RH, Lee SH, Wang Y, Park S-R, Kim ST. 2017. Global transcriptome profiling of *Xanthomonas oryzae* pv. *oryzae* under in planta growth and *in vitro* culture conditions. Plant Pathol J 33:458–466. doi:10.5423/PPJ.OA.04.2017.0076 29018309 PMC5624488

[24] Liao Z-X, Ni Z, Wei X-L, Chen L, Li J-Y, Yu Y-H, Jiang W, Jiang B-L, He Y-Q, Huang S. 2019. Dual RNA-seq of *Xanthomonas oryzae* pv. *oryzicola* infecting rice reveals novel insights into bacterial-plant interaction. PLoS One 14:e0215039. doi:10.1371/journal.pone.0215039 30995267 PMC6469767

[25] Luneau JS, Baudin M, Quiroz Monnens T, Carrère S, Bouchez O, Jardinaud M-F, Gris C, François J, Ray J, Torralba B, Arlat M, Lewis JD, Lauber E, Deutschbauer AM, Noël LD, Boulanger A. 2022. Genome-wide identification of fitness determinants in the *Xanthomonas campestris* bacterial pathogen during early stages of plant infection. New Phytol 236:235–248. doi:10.1111/nph.18313 35706385 PMC9543026

[26] Lavín JL, Kiil K, Resano O, Ussery DW, Oguiza JA. 2007. Comparative genomic analysis of two-component regulatory proteins in *Pseudomonas syringae*. BMC Genomics 8:397. doi:10.1186/1471-2164-8-397 17971244 PMC2222644

[27] Chatterjee A, Cui Y, Yang H, Collmer A, Alfano JR, Chatterjee AK. 2003. GacA, the response regulator of a two-component system, acts as a master regulator in *Pseudomonas syringae* pv. *tomato* DC3000 by controlling regulatory RNA, transcriptional activators, and alternate sigma factors. Mol Plant Microbe Interact 16:1106–1117. doi:10.1094/MPMI.2003.16.12.1106 14651344

[28] Deng X, Liang H, Chen K, He C, Lan L, Tang X. 2014. Molecular mechanisms of two-component system RhpRS regulating type III secretion system in *Pseudomonas syringae*. Nucleic Acids Res 42:11472–11486. doi:10.1093/nar/gku865 25249629 PMC4191427

[29] Fishman MR, Zhang J, Bronstein PA, Stodghill P, Filiatrault MJ. 2018. Ca^2+^-induced two-component system CvsSR regulates the type III secretion system and the extracytoplasmic function sigma factor AlgU in *Pseudomonas syringae* pv. *tomato* DC3000. J Bacteriol 200:e00538-17. doi:10.1128/JB.00538-17 29263098 PMC5809696

[30] Shao X, Tan M, Xie Y, Yao C, Wang T, Huang H, Zhang Y, Ding Y, Liu J, Han L, Hua C, Wang X, Deng X. 2021. Integrated regulatory network in *Pseudomonas syringae* reveals dynamics of virulence. Cell Rep 34:108920. doi:10.1016/j.celrep.2021.108920 33789108

[31] Sreedharan A, Penaloza-Vazquez A, Kunkel BN, Bender CL. 2006. CorR regulates multiple components of virulence in *Pseudomonas syringae* pv. *tomato* DC3000. Mol Plant Microbe Interact 19:768–779. doi:10.1094/MPMI-19-0768 16838789

[32] Lovelace AH, Smith A, Kvitko BH. 2018. Pattern-triggered immunity alters the transcriptional regulation of virulence-associated genes and induces the sulfur starvation response in *Pseudomonas syringae* pv. *tomato* DC3000. Mol Plant Microbe Interact 31:750–765. doi:10.1094/MPMI-01-18-0008-R 29460676

[33] Honda T, Yu S, Mai D, Baumgart L, Babnigg G, Yoshikuni Y. 2025. CRAGE-RB-PI-seq enables transcriptional profiling of rhizobacteria during plant-root colonization. Microbiology. doi:10.1101/2024.11.19.624340

[34] Wang H, Smith A, Lovelace A, Kvitko BH. 2022. In planta transcriptomics reveals conflicts between pattern-triggered immunity and the AlgU sigma factor regulon. PLoS One 17:e0274009. doi:10.1371/journal.pone.0274009 36048876 PMC9436044

[35] De Francesco A, Lovelace AH, Shaw D, Qiu M, Wang Y, Gurung F, Ancona V, Wang C, Levy A, Jiang T, Ma W. 2022. Transcriptome profiling of “*Candidatus* Liberibacter asiaticus” in Citrus and Psyllids. Phytopathology 112:116–130. doi:10.1094/PHYTO-08-21-0327-FI 35025694

[36] Yang S, Lovelace AH, Yuan Y, Nie H, Chen W, Gao Y, Bo W, Nagel DH, Pang X, Ma W. 2025. A witches’ broom phytoplasma effector induces stunting by stabilizing a bHLH transcription factor in *Ziziphus jujuba* plants. New Phytol 247:249–264. doi:10.1111/nph.70172 40348577 PMC12138163

[37] Fishman MR, Filiatrault MJ. 2019. Prevention of surface-associated calcium phosphate by the *Pseudomonas syringae* two-component system CvsSR. J Bacteriol 201:e00584-18. doi:10.1128/JB.00584-18 30617243 PMC6416903

[38] Smith A, Lovelace AH, Kvitko BH. 2018. Validation of RT-qPCR approaches to monitor *Pseudomonas syringae* gene expression during infection and exposure to pattern-triggered immunity. MPMI 31:410–419. doi:10.1094/MPMI-11-17-0270-TA 29436925

[39] Bolger AM, Lohse M, Usadel B. 2014. Trimmomatic: a flexible trimmer for Illumina sequence data. Bioinformatics 30:2114–2120. doi:10.1093/bioinformatics/btu170 24695404 PMC4103590

[40] Wang N, Han N, Tian R, Chen J, Gao X, Wu Z, Liu Y, Huang L. 2021. Role of the type VI secretion system in the pathogenicity of *Pseudomonas syringae* pv. *actinidiae*, the causative agent of kiwifruit bacterial canker. Front Microbiol 12. doi:10.3389/fmicb.2021.627785 PMC7933208 33679650

[41] Kim N, Kim JJ, Kim I, Mannaa M, Park J, Kim J, Lee H-H, Lee S-B, Park D-S, Sul WJ, Seo Y-S. 2020. Type VI secretion systems of plant-pathogenic *Burkholderia glumae* BGR1 play a functionally distinct role in interspecies interactions and virulence. Mol Plant Pathol 21:1055–1069. doi:10.1111/mpp.12966 32643866 PMC7368126

[42] Luu RA, Schomer RA, Brunton CN, Truong R, Ta AP, Tan WA, Parales JV, Wang Y-J, Huo Y-W, Liu S-J, Ditty JL, Stewart V, Parales RE. 2019. Hybrid two-component sensors for identification of bacterial chemoreceptor function. Appl Environ Microbiol 85:e01626-19. doi:10.1128/AEM.01626-19 31492670 PMC6821969

[43] King EO, Ward MK, Raney DE. 1954. Two simple media for the demonstration of pyocyanin and fluorescin. J Lab Clin Med 44:301–307. 13184240

[44] Langmead B, Salzberg SL. 2012. Fast gapped-read alignment with Bowtie 2. Nat Methods 9:357–359. doi:10.1038/nmeth.1923 22388286 PMC3322381

[45] Pertea M, Kim D, Pertea GM, Leek JT, Salzberg SL. 2016. Transcript-level expression analysis of RNA-seq experiments with HISAT, StringTie and Ballgown. Nat Protoc 11:1650–1667. doi:10.1038/nprot.2016.095 27560171 PMC5032908

[46] Love MI, Huber W, Anders S. 2014. Moderated estimation of fold change and dispersion for RNA-seq data with DESeq2. Genome Biol 15:550. doi:10.1186/s13059-014-0550-8 25516281 PMC4302049

[47] Luo W, Friedman MS, Shedden K, Hankenson KD, Woolf PJ. 2009. GAGE: generally applicable gene set enrichment for pathway analysis. BMC Bioinformatics 10:161. doi:10.1186/1471-2105-10-161 19473525 PMC2696452

