# Supplementary material for: The two-component regulator CvsR has a small core regulon in planta and modulates Pseudomonas syringae global gene expression with some overlap to the pattern-triggered immunity stimulon response
Source: Microbiol Spectr. 2026 Jun 22;14(8):e04124-25. doi: 10.1128/spectrum.04124-25 (PMC13436366; doi:10.1128/spectrum.04124-25)
Supplement: Supplementary file 1 [file spectrum.04124-25-s0001.docx]

**Supporting Materials**

**Table S1.1 Sequences mapping rate to *Pseudomonas syringae* pv *tomato* DC3000 genome**

**Table S1.2 Sequences mapping rate to *Arabidopsis thaliana* col-0 genome**

**Table S2.1 significant DEGs between flg22 and mock treatment in wild-type strains (WF/WM)**

**Table S2.2 significant DEGs between flg22 and mock treatment in Δ*cvsR* strains (CF/CM)**

**Table S2.3 significant DEGs of Δ*cvsR* and wild-type strains under mock treatments (CM/WM)**

**Table S2.4 significant DEGs of Δ*cvsR* and wild-type strains under flg22 treatments (CF/WF)**

**Table S3. Concordantly regulated overlapping DEGs between the Lovelace and Fishman datasets**

**Figure S1 Pre-induced pattern-triggered immunity (PTI) protects plant** **tissue from infection and Δ*cvsR* showed no significant different of in planta bacterial population to wild type strain** (A) Growth assay of *Pseudomonas syringae* pv. t*omato* DC3000 (*Pto* DC3000) WT in 1 µM flg22 treated tissue of 4.5-week-old *Arabidopsis thaliana* col-0 for PTI induction at 16h and 20h prior to inoculation at OD600= 0.8 (10^9^ CFU/mL) and harvested at 5 and 24 hpi. different color indicated flg22 pre-treatment at 16 h or 20 h prior to bacterial inoculation (B) Growth assay of *Pto* DC3000 WT and Δ*cvsR* strain in 1 µM flg22 or 0.1% DMSO treated tissue 16h prior to the inoculum at OD600= 0.8 (10^9^ CFU/mL) and harvested at 5 and 24 hpi. Bars represent means and standard deviations calculated from individual replicate (n = 4). Asterisks indicate statistically significant differences for given comparison (16h vs. 20h flg22 pretreatment or WT vs. Δ*cvsR*) (2 tailed t.test, *P* < 0.05 ); ns=non-significant. (C) Symptoms of infected pretreated leaves by *Pto* DC3000 at 24/72 hpi with the number of leaves that exhibited the symptoms represented above out of the total number of leaves recorded (n=8). Scale bar = 1.0 cm.

**Figure S2 Expression heatmap representing sample-to-sample distances calculated from the variance-stabilizing transformation of RNA-seq count data across all genes.** Hierarchical clustering of 18 RNA-seq samples provides an overview of relationships among genotypes, with sample proximity indicated by the intensity of square colors. Blue areas correspond to closely related samples with similar expression profiles, while yellow regions highlight greater sample-to-sample dissimilarity.

**Figure S3. Raw read counts per sample of CvsR direct regulon.** The y-axis represents the read counts and the x-axis represents sample type (strain/treatment). Each boxplot shows the distribution of read counts for three biological replicates (n=3) within each sample type.

**Figure S4 Expression of bacterial sulfur metabolism genes.** (A) Normalized reads of gene listed in sulfate metabolism pathway of *P. syringae* pv. *tomato* DC3000 in each strain/condition: $\Delta$*cvsR* (C), wild type (W), flg22 treatment (F), and mock control (M). (B) log_2_ fold change in mean normalized gene counts of $\Delta$*cvsR* relative to wildtype of in vitro (KB), naïve tissue (mock), and PTI tissue (flg22). Asterisks indicate significant in changes for $\Delta$*cvsR* relative to wild-type (2 tailed t.test, *P* < 0.05).

**Figure S5. Expression of bacterial chemotaxis genes.** (A) Normalized reads of gene listed in bacterial chemotaxis pathway of *P. syringae* pv. *tomato* DC3000 in each strain/condition: $\Delta$*cvsR* (C), wild type (W), flg22 treatment (F), and mock control (M). (B) log_2_ fold change in mean normalized gene counts of $\Delta$*cvsR* relative to wildtype of in vitro (KB), naïve tissue (mock), and PTI tissue (flg22). Asterisks indicate significant in changes for $\Delta$*cvsR* relative to wildtype (2 tailed t.test, *P* < 0.05).

**Figure S6. Expression of bacterial flagella assembly genes.** (A) Normalized reads of gene listed in bacterial flagella assembly pathway of *P. syringae* pv. *tomato* DC3000 in each strain/condition: $\Delta$*cvsR* (C), wild type (W), flg22 treatment (F), and mock control (M). (B) log_2_ fold change in mean normalized gene counts of $\Delta$*cvsR* relative to wildtype of in vitro (KB), naïve tissue (mock), and PTI tissue (flg22). Asterisks indicate significant in changes for $\Delta$*cvsR* relative to wildtype (2 tailed t.test, *P* < 0.05).

**Figure S7. Reverse transcription-quantitative polymerase chain reaction (RT-qPCR) analysis of *Pseudomonas syringae* pv. t*omato* DC3000 gene expression during early exposure to pattern-triggered immunity (PTI) relative to naïve host infection of *A. thaliana*.** Expression of two sulfate metabolism–related genes (*sfbp* and *sbp*) in bacteria exposed to PTI relative to expression in bacteria during naïve host infection (PTI versus naïve), normalized against the expression of the reference gene *recA*. Bars represent mean log₂ normalized relative quantity (NRQ) and standard error calculated from independent biological replicate samples (n = 3). Different letters indicate statistically significant differences between conditions (2 tailed t.test, *P* < 0.05). Δ*cvsR* (C), wild type (W), flg22 treatment (F), and mock control (M).
